# Circadian Rhythms of Locomotor Activity Mediated by *Cryptochrome 2* and *Period 1* Genes in the Termites *Reticulitermes chinensis* and *Odontotermes formosanus*

**DOI:** 10.3390/insects15010001

**Published:** 2023-12-19

**Authors:** Yongyong Gao, Huan Xu, Bao Jia, Yutong Liu, Ali Hassan, Qiuying Huang

**Affiliations:** 1 Hubei Insect Resources Utilization and Sustainable Pest Management Key Laboratory, College of Plant Science and Technology, Huazhong Agricultural University, Wuhan 430070, China; yongyonggao@yau.edu.cn (Y.G.); huanxu@webmail.hzau.edu.cn (H.X.); 331615265@163.com (B.J.); liuyutong@webmail.hzau.edu.cn (Y.L.); alihassan2771@gmail.com (A.H.); 2 Research and Development Centre of Ecological and Sustainable Application of Microbial Industry of the Loess Plateau in Shaanxi Province, College of Life Science, Yan’an University, Yan’an 716000, China; 3 Key Laboratory of Termite Control of Ministry of Water Resources, Huazhong Agricultural University, Wuhan 430070, China

**Keywords:** circadian rhythms, termites, circadian clock, *cryptochrome 2*, *period 1*

## Abstract

**Simple Summary:**

Almost all insects demonstrate a wide variety of behavioral, physiological, biochemical and molecular circadian rhythms during their natural selection process. Some examples of the circadian rhythm in insects include the sleep–wake cycle, foraging time, migration and hormone fluctuation. Although the circadian rhythms of activities have been studied in several termite species, the molecular mechanisms of circadian rhythms in termites are unclear. In this study, we observed that, even in complete darkness, the termites *Reticulitermes chinensis* and *Odontotermes formosanus* showed clear circadian rhythms of locomotor activity. Knockdown of the clock genes *cryptochrome 2* (*Cry2*) and *period 1* (*Per1*) impaired the circadian rhythms of locomotor activity in constant darkness in the two termite species. We verified that locomotor activity in subterranean termites is controlled by the circadian clock. Thus, this study contributes to a better understanding of circadian clock mechanisms in subterranean insects.

**Abstract:**

Locomotor activity rhythms are crucial for foraging, mating and predator avoidance in insects. Although the circadian rhythms of activity have been studied in several termite species, the molecular mechanisms of circadian rhythms in termites are still unclear. In this study, we found that two termite species, *R. chinensis* and *O. formosanus*, exhibited clear circadian rhythms of locomotor activity in constant darkness along with rhythmically expressed core clock genes, *Cry2* and *Per1*. The knockdown of *Cry2* or *Per1* expression in the two termite species disrupted the circadian rhythms of locomotor activity and markedly reduced locomotor activity in constant darkness, which demonstrates that *Cry2* and *Per1* can mediate the circadian rhythms of locomotor activity in termites in constant darkness. We suggest that locomotor activity in subterranean termites is controlled by the circadian clock.

## 1. Introduction

Almost all insects demonstrate a wide variety of behavioral, physiological, molecular and biochemical circadian rhythms during their natural selection process [1]. In insects, circadian rhythms are observed in various forms, such as the sleep–wake cycle, foraging/mating time, migration, gene expression, hormone fluctuation and so on [2,3,4,5]. Over the past 30 years, the molecular mechanisms of circadian rhythms in insects have been extensively investigated [6]. At present, *Drosophila* is the primary model used in research on the molecular mechanisms of the circadian clock in insects [7,8,9]. In *Drosophila*, the endogenous circadian clock is driven primarily by positive and negative feedback loops that involve a series of circadian clock genes, such as *period* (*Per*), *timeless* (*Tim*), *cryptochrome* (*Cry*), *cycle* (*Cyc*), *clock* (*Clk*), *double-time* (*Dbt*), *shaggy* (*Sgg*), *vrille* (*Vri*), *clockwork-orange* (*Cwo*), etc. [10,11,12,13,14].

In insects and mammals, it is well recognized that *Cry*, *Per* and *Tim* play a vital role in the endogenous circadian clock pathway [15,16,17]. In the cockroach *Blattella germanica*, the knockdown of *Cry2* severely disrupted the circadian rhythms of locomotor activity even in constant darkness (DD) [18]. Under a 12 h light:12 h dark cycle (LD), mice exhibited clear circadian rhythms of locomotor activity, whereas with a double knockout of *Cry1*/*Cry2* in mice they exhibited no clear circadian rhythms of locomotor activity [19]. Similarly, mosquitoes show a rhythmicity of swarming to attract females for mating at certain times of the day [20], but knocking down the circadian clock genes *Per* and *Tim* disrupts male swarming behavior and limits their mating success [5]. In the cricket *Gryllus bimaculatus*, the knockdown of *Per* impaired the circadian rhythm of locomotor activity under DD conditions [21]. Thus, these examples indicate that the circadian clock genes *Cry*, *Per* and *Tim* can regulate the circadian rhythms of activities in insects and mammals. Notably, a recent study verified that *Tim* is lost in some social insects (nearly all termites) [22]. In addition, light intensity, temperature and social interactions regulate the circadian rhythms of behavior and physiology in insects [23,24,25,26,27]. Although the circadian rhythms of activities have been studied in several termite species (*R*. *speratus*, *Macrotermes bellicosus*, etc.) [28,29,30], the molecular mechanisms of circadian rhythm activity in subterranean termites are unclear.

The termites *Reticulitermes chinensis* and *Odontotermes formosanus* are widely distributed in China, especially in the area south of the Yangtze River [31]. These two termite species make excellent candidates for studying circadian rhythms due to their irrelevant visual cues [32,33]. In addition, the study of circadian rhythms in termites provides insight into the rhythmicity of subterranean insects and mammals. In order to investigate the behavioral and molecular mechanisms of activity rhythms in subterranean termites, we first tested the circadian rhythms of locomotor activity in the two termite species under different photoperiods. Second, we assessed the temporal expression patterns of clock genes in the heads of two termite species. Finally, we used RNA interference to analyze the influence of the clock genes *Cry2* and *Per1* on the circadian rhythms of locomotor activity in the two termite species.

## 2. Materials and Methods

### 2.1. Experimental Termites

We collected the termites *R. chinensis* and *O. formosanus* from Shizi Hill (Wuhan, China). The number of termite colonies used in this study is shown in Supplementary Tables S1 and S2. We transported these colonies of termites to the laboratory and then placed them in plastic boxes. Before the experiments, the termites were fed with pieces of moist filter paper for 3 days. Worker termites were used as experimental subjects in these experiments. The conditions for rearing the worker termites were as follows: 25 ± 1 °C and 70 ± 5% relative humidity (RH) in full darkness.

### 2.2. Measurement of Locomotor Activity

**Figure 1:**
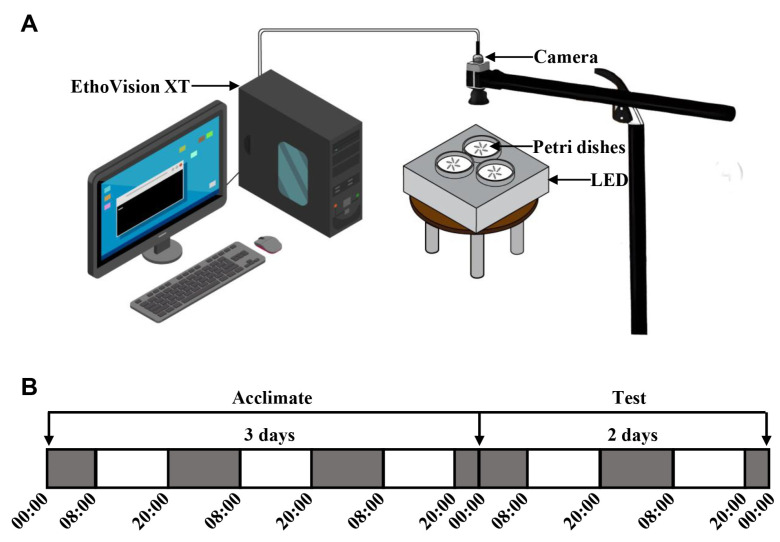
Experimental settings and tracking method for the termites. (**A**) Experimental setup of the quantitative system for analyzing behavior of termites. The video recordings were converted to mpeg format for analysis of the parameters using the EthoVision video tracking system. (**B**) Schematic diagram of the timeline for the behavioral assay.

Termites were transferred to 9 cm Petri dishes (six worker termites per dish) with moist filter paper and then sealed with sealing film (it has been discovered in laboratories that moist filter paper can remain wet for more than 10 days). Before the experiments, termites were randomly divided into three different groups. One group was acclimated to constant darkness (DD) for 3 days, and the other two groups were acclimated to the light/dark cycle (LD, 12 L:12 D, illumination time 08:00–20:00 (Beijing Time, China); light intensity, 100 lux; irradiance, 0.62 W/m^−2^) or constant light (LL, light intensity, 100 lux; irradiance, 0.62 W/m^−2^) for 3 days, respectively (Figure 1A,B). During each experiment, we used a video camera with an infrared module (acA1920-40gc, Basler, Germany) to record the circadian rhythms of the locomotor activity of worker termites. The camera captured video at 25 frames/s, and video analysis was conducted using the Noldus EthoVision tracking system (EthoVision XT 14, Wageningen, Netherlands) [32,33,34]. The distances moved by worker termites were measured in different photoperiods (DD, LD or LL) for 48 h (Figure 1A,B). The conditions of the trials were as follows: 25 ± 1 °C and 70 ± 5% RH in infrared illumination [35].

### 2.3. Expression Patterns of Clock Genes

Expression patterns of clock genes (*Cry2* and *Per1*) were observed at different times of the day (00:00, 04:00, 08:00, 12:00, 16:00 and 20:00) (Beijing Time, China) under DD and LD conditions by qRT-PCR. We extracted the total RNA from the heads of 10 worker termites using RNAiso Plus (Takara, Code No: 9109, Dalian, China). Then, we synthesized cDNA templates using total RNA at the concentration of one microgram according to the instructions of the PrimeScript^TM^ RT Reagent Kit with gDNA Eraser (Takara, Code No: RR047A, Dalian, China). Next, qRT-PCR was performed with the synthesized cDNA templates, gene-specific primers and SYBR Green Master Mix (High Rox Plus) (YEASEN, Code No: 11203ES08, Shanghai, China) using the QuantStudio 6&7 Flex Real-Time PCR System (Applied Biosystems, Life Technologies Italia, MA, USA). Finally, mRNA levels were quantified using three genes (*β-actin*, *Hsp 70* or *NADH*) as the reference genes [32,35]. The primer sequences used for qRT-PCR are presented in Supplementary Table S3. The qRT-PCR data for the two clock genes *Cry2* and *Per1* were calculated via the 2^−ΔΔCT^ method to analyze them [36].

### 2.4. Synthesis of dsRNA and Microinjection

Double-stranded *Cry2* (ds*Cry2*) and *Per1* (ds*Per1*) were synthesized with the T7 transcription kit (Thermo Fisher Scientific, Code No: AM1354, MA, USA) according to the manufacturer’s instructions. Firstly, PCR was carried out using the plasmids as templates in combination with specific primers (See Supplementary Table S4). Subsequently, with the purified PCR products as templates, T7 RNA Polymerase was used to generate dsRNA in transcription reactions. Next, dsRNA was dissolved in diethyl pyrocarbonate water and quantified by the NanoDrop 2000 Spectrophotometer (Thermo Fisher Scientific, MA, USA). The dsRNA was subjected to 1% agarose gel, and the dsRNA solution was stored at −80 °C.

Two micrograms of dsRNA solution were injected into the side of the thorax in worker termites using a sterilized microinjector (World Precision Instruments, SYS-PV820, Florida, USA) [37,38]. Termites were placed on moist filter paper within 9 cm Petri dishes after dsRNA injection. Ten worker termites were collected for measuring the mRNA of *Cry2* or *Per1* three days and five days after injection. In addition, three days after injection, the locomotor activities of worker termites were recorded for 48 h. An equivalent solution of double-stranded *green fluorescent protein* (ds*GFP*) was injected in control groups [39].

### 2.5. Statistical Analysis

The rhythms of locomotor activity over a 48 h period were determined using the cosinor procedure (http://www.circadian.org/softwar.html, accessed on 13 November 2023) [40]. IBM SPSS Statistics 19.0 software was used to analyze the metric data. The normal distribution of the metric data was tested using the Shapiro–Wilk method. One-way analysis of variance (*ANOVA*) and Tukey’s HSD test were used to analyze the temporal expression levels of genes and walking distances. The abnormal distribution of the metric data was tested using the Wilcoxon signed-rank test and Mann–Whitney test. The Wilcoxon rank-sum test was used to analyze the expression of the targeted genes *Cry2* or *Per1* after injection. Locomotor activity during the subjective day and subjective night was evaluated using the Mann–Whitney test. The significance level was set as *p* < 0.05.

## 3. Results

### 3.1. Effects of Different Photoperiods on the Circadian Rhythms of Locomotor Activity in the Two Termite Species

**Figure 2:**
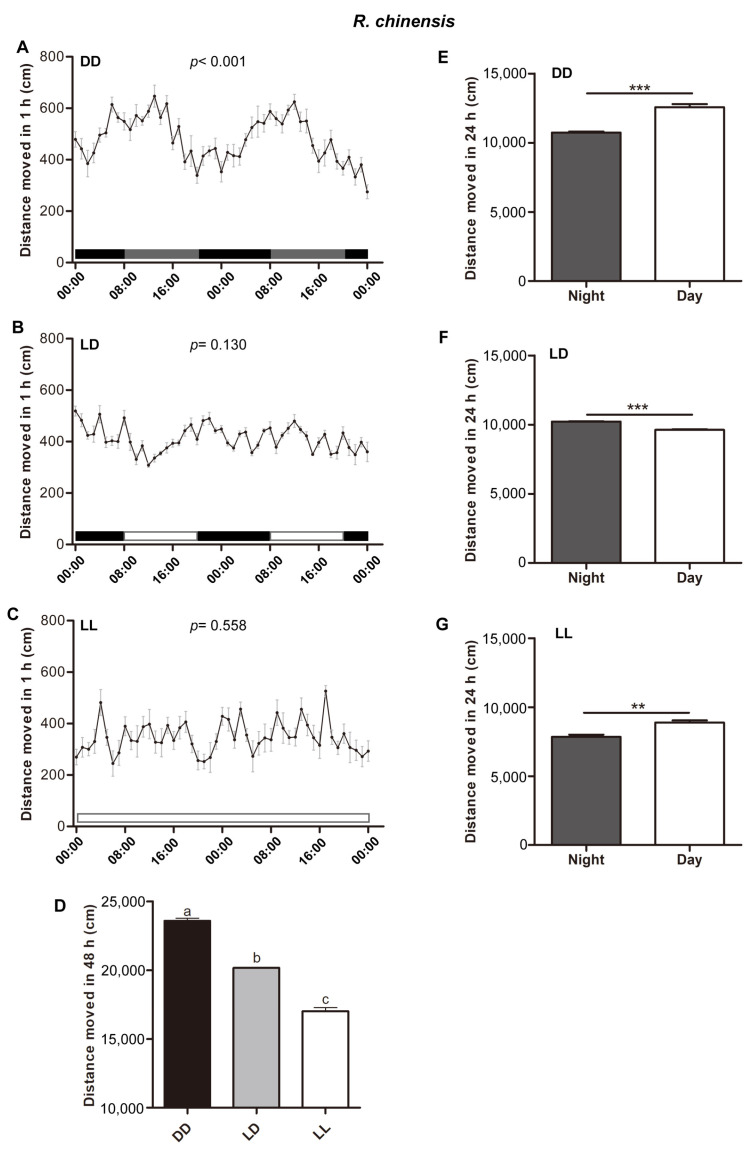
The circadian rhythms of locomotor activity in the termite *R. chinensis* under different photoperiods. (**A**–**C**) Locomotor activity of the termite *R. chinensis* under DD (n = 10) (**A**), LD (n = 9) (**B**) and LL (n = 9) (**C**) photoperiod conditions. The data in the figures are mean ± SEM, and the circadian rhythms of locomotor activity were analyzed using the cosinor procedure (**A**–**C**). (**D**) The total walking distances of the termite *R. chinensis* during a 48 h experimental period. The data in the figures are mean ± SEM, and different letters express significant differences according to Tukey’s HSD test (*p* < 0.05) (**D**). (**E**–**G**) The total walking distances of the termite *R. chinensis* in subjective day and subjective night under DD (**E**), LD (**F**) and LL (**G**) photoperiod conditions. The data represent mean ± SEM. Asterisks indicate significant differences determined by the Mann–Whitney test (** *p* < 0.01; *** *p* < 0.001) (**E**–**G**). The horizontal bars represent the light conditions during the experiment. White boxes represent light and black and gray boxes represent dark in (**A**–**C**).

**Figure 3:**
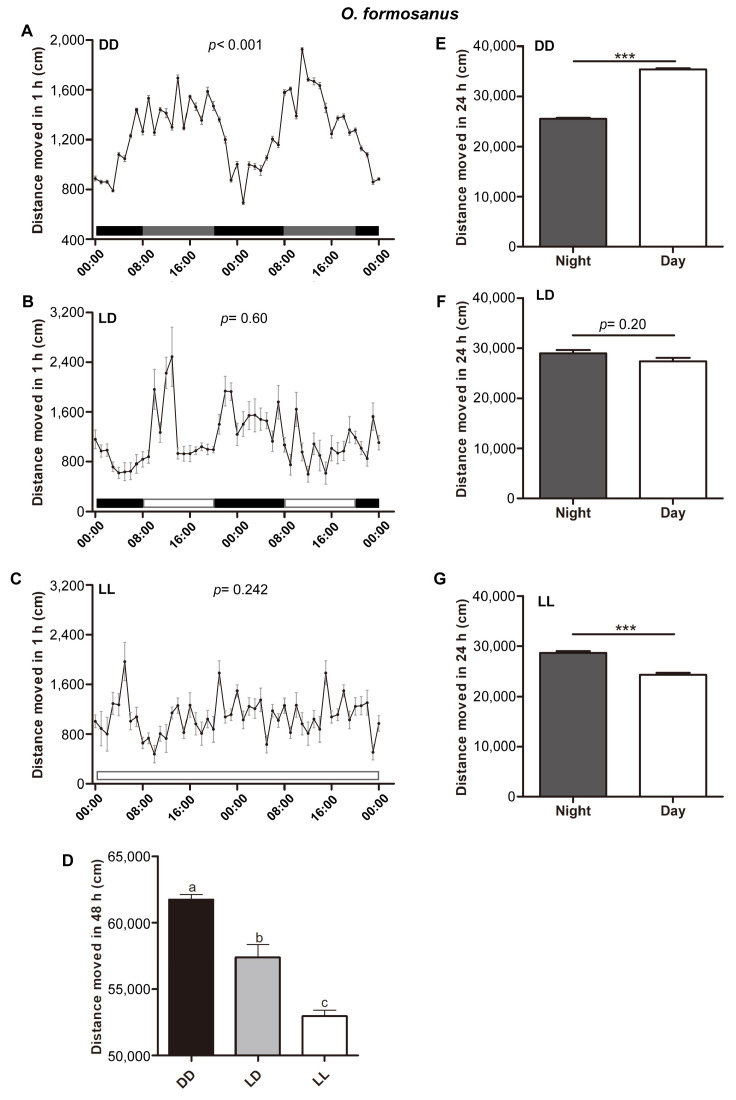
The circadian rhythms of locomotor activity in the termite *O. formosanus* under different photoperiods. (**A**–**C**) Locomotor activity of the termite *O. formosanus* under DD (n = 10) (**A**), LD (n = 9) (**B**) and LL (n = 9) (**C**) photoperiod conditions. The data in the figures are mean ± SEM, and the circadian rhythms of locomotor activity were analyzed using the cosinor procedure (**A**–**C**). (**D**) The total walking distances of the termite *O. formosanus* during a 48 h experimental period. The data in the figures are mean ± SEM, and different letters express significant differences according to Tukey’s HSD test (*p* < 0.05) (**D**). (**E**–**G**) The total walking distances of the termite *O. formosanus* in subjective day and subjective night under DD (**E**), LD (**F**) and LL (**G**) photoperiod conditions. The data represent mean ± SEM. Asterisks indicate significant differences determined by the Mann–Whitney test (*** *p* < 0.001) (**E**–**G**).

We examined the effects of different photoperiods on the circadian rhythms of locomotor activity in the two termites *R. chinensis* and *O. formosanus*. We found that the termite *R. chinensis* exhibited a significant circadian rhythm of locomotor activity under DD conditions (*p* < 0.001) (Figure 2A). Similarly, the termite *O. formosanus* also exhibited a significant circadian rhythm of locomotor activity under DD conditions (*p* < 0.001) (Figure 3A). At the same time, the circadian periods of the two termites *R. chinensis* and *O. formosanus* were close to 24 h under DD conditions. However, under LD and LL conditions, the circadian rhythms of locomotor activity were abolished in the two termites *R. chinensis* (LD, *p* = 0.130; LL, *p* = 0.558) (Figure 2B,C) and *O. formosanus* (LD, *p* = 0.60; LL, *p* = 0.242) (Figure 3B,C).

We also found that locomotor activity showed significant changes under different photoperiods in the two termites *R. chinensis* (*ANOVA*, F_(2,25)_ = 285.21, *p* < 0.001) (Figure 2D) and *O. formosanus* (*ANOVA*, F_(2,25)_ = 46.91, *p* < 0.001) (Figure 3D), and locomotor activity under DD conditions was significantly higher than that under LD and LL conditions in the two termite species. Moreover, locomotor activity during the subjective day was significantly higher than during the subjective night under DD conditions in the two termites *R. chinensis* (*Mann–Whitney test*, *Z* = −3.78, *df* = 20, *p* < 0.001) (Figure 2E) and *O. formosanus* (*Mann–Whitney test*, Z = −3.78, *df* = 20, *p* < 0.001) (Figure 3E). Under LD conditions, locomotor activity during the subjective day was significantly lower than during the subjective night in the termite *R. chinensis* (*Mann–Whitney test*, *Z* = −3.58, *df* = 18, *p* < 0.001) (Figure 2F), but there was no significant difference in daily active intensity in the termite *O. formosanus* (*Mann–Whitney test*, *Z* = −1.28, *df* = 18, *p* = 0.20) (Figure 3F). Under LL conditions, locomotor activity during the subjective day was significantly higher than during the subjective night in the termite *R. chinensis* (*Mann–Whitney test*, *Z* = −3.14, *df* = 18, *p* < 0.01) (Figure 2G), but locomotor activity during the subjective day was significantly lower than during the subjective night in the termite *O. formosanus* (*Mann–Whitney test*, *Z* = −3.58, *df* = 18, *p* < 0.001) (Figure 3G).

### 3.2. Cry2 and Per1 Temporal Expression under DD Conditions in the Two Termite Species

**Figure 4:**
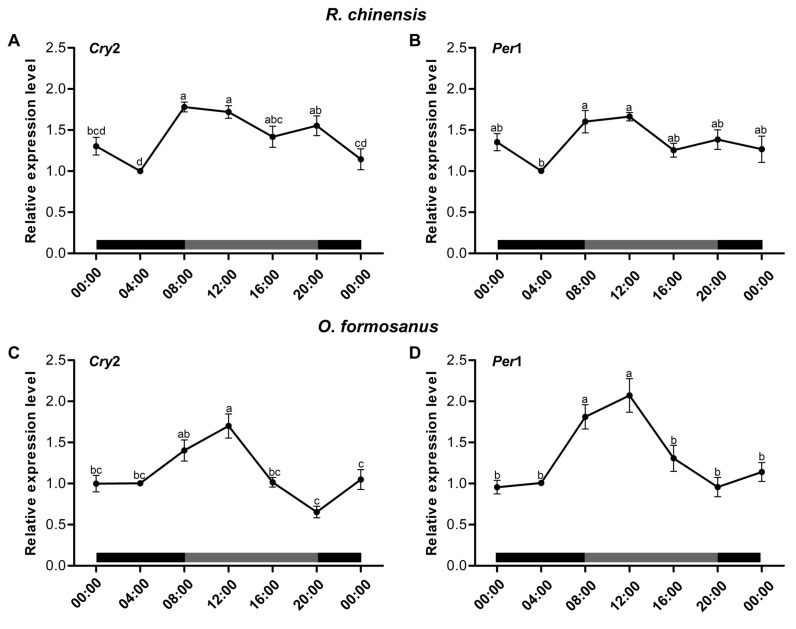
The patterns of *Cry2* and *Per1* gene expression in two termite species. (**A**) The patterns of *Cry2* expression in the termite *R. chinensis* under DD conditions (n = 8). (**B**) The patterns of *Per1* expression in the termite *R. chinensis* under DD conditions (n = 8). (**C**) The patterns of *Cry2* expression in the termite *O. formosanus* under DD conditions (n = 8). (**D**) The patterns of *Per1* expression in the termite *O. formosanus* under DD conditions (n = 8). The data in the figures are the mean ± SEM, and different letters express significant differences according to Tukey’s HSD test (*p* < 0.05). The mRNA levels were normalized relative to the two termite species collected at 04:00. Gray and black bars represent subjective day and subjective night, respectively.

We assayed head *Cry2* and *Per1* mRNA abundance over time in the two termite species by qRT-PCR in DD and LD conditions. Under DD conditions, we found that the expression of *Cry2* and *Per1* in the termite *R. chinensis* showed significant differences at different times of the day (*ANOVA*, *Cry2*, F_(6,49)_ = 8.67, *p* < 0.001; *Per1*, F_(6,49)_ = 4.42, *p* < 0.001), and the expression of *Cry2* and *Per1* in *R. chinensis* showed peak levels at 08:00–12:00 and its lowest level at 04:00 (Figure 4A,B). In the termite *O. formosanus*, we also found that the expression of *Cry2* and *Per1* showed significant differences at different times of the day (*ANOVA*, *Cry2*, F_(6,49)_ = 11.18, *p* < 0.001; *Per1*, F_(6,49)_ = 11.40, *p* < 0.001), and the expression of *Cry2* and *Per1* in *O. formosanus* showed peak levels at 12: 00 and its lowest level at 20:00 (Figure 4C,D). In addition, under LD conditions, we found that the expression of *Cry2* and *Per1* in the termites *R. chinensis* (*ANOVA*, *Cry2*, F_(6,56)_ = 0.56, *p* = 0.764; *Per1*, F_(6,56)_ = 1.82, *p* = 0.112) and *O. formosanus* (*ANOVA*, *Cry2*, F_(6,56)_ = 1.95, *p* = 0.089; *Per1*, F_(6,56)_ = 2.24, *p* = 0.052) showed no significant differences at different times of the day (Supplementary Figure S1).

### 3.3. Cry2 and Per1 Knockdown Disrupted the Circadian Rhythms of Locomotor Activity under DD Conditions in the Two Termite Species

**Figure 5:**
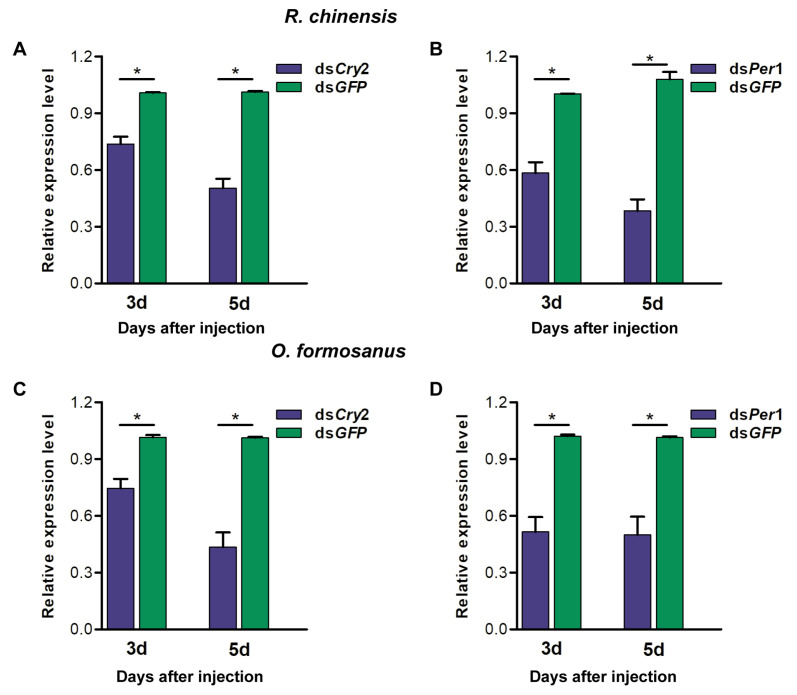
Injection of dsCry2 and dsPer1 reduced mRNA levels of the genes *Cry2* and *Per1* in the two termite species. (**A**,**B**) The mRNA levels of *Cry2* (3 days: n = 7; 5 days: n = 4) (**A**) and *Per1* (3 days: n = 7; 5 days: n = 4) (**B**) in the termite *R. chinensis* 3 days and 5 days after injecting dsRNA. (**C**,**D**) The mRNA levels of *Cry2* (3 days: n = 9; 5 days: n = 4) (**C**) and *Per1* (3 days: n = 9; 5 days: n = 4) (**D**) in the termite *O. formosanus* 3 days and 5 days after injecting dsRNA. The bars represent mean ± SEM. Asterisks indicate significant differences determined by the Wilcoxon signed-rank test (* *p* < 0.05).

The injection of ds*Cry2* or ds*Per1* caused a significant decrease in the *Cry2* or *Per1* mRNA level in the head of the termite *R. chinensis* (*Wilcoxon signed-rank test*, 3 days: *Cry2*, *Z* = −2.36, n = 7, *p* < 0.05; *Per1*, *Z* = −2.36, n = 7, *p* < 0.05; 5 days: *Cry2*, *Z* = −2.31, n = 4, *p* < 0.05; *Per1*, *Z* = −2.31, n = 4, *p* < 0.05) (Figure 5A,B), suggesting that *Cry2* and *Per1* were significantly knocked down 3 days and 5 days after injection. Similarly, the injection of ds*Cry2* or ds*Per1* caused a significant decrease in *Cry2* or *Per1* mRNA level in the head of the termite *O. formosanus* (*Wilcoxon signed-rank test*, 3 days: *Cry2*, *Z* = −2.67, n = 9, *p* < 0.05; *Per1*, *Z* = −2.37, n = 7, *p* < 0.05; 5 days: *Cry2*, *Z* = −1.83, n = 4, *p* < 0.05; *Per1*, *Z* = −1.83, n = 4, *p* < 0.05) (Figure 5C,D), suggesting that *Cry2* and *Per1* were significantly knocked down 3 days and 5 days after injection.

**Figure 6:**
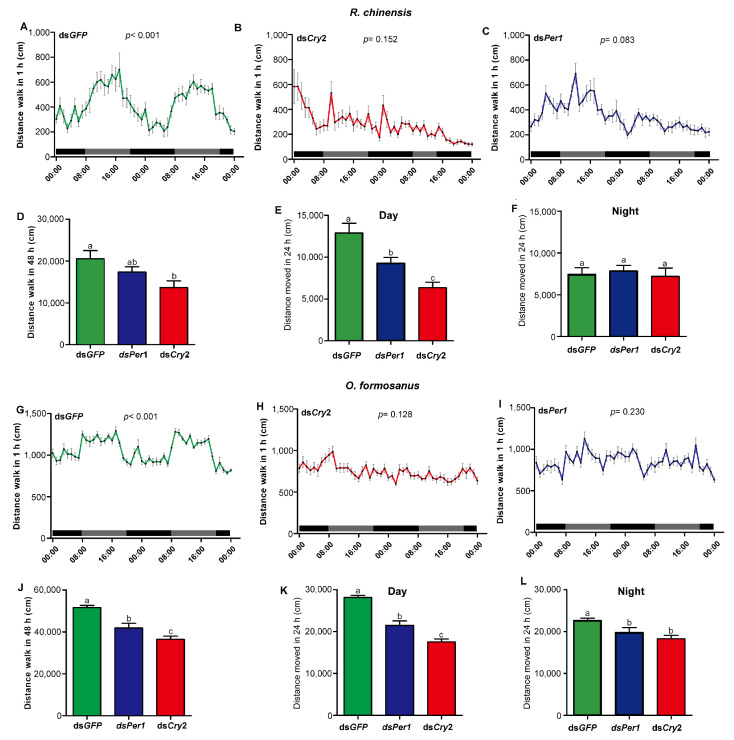
Knockdown of *Cry2* and *Per1* disrupted the circadian rhythms of locomotor activities in the two termite species. (**A**–**C**) The circadian rhythms of locomotor activities of the termite *R. chinensis* injected with ds*GFP* (n = 12) (**A**), ds*Cry2* (n = 12) (**B**), or ds*Per1* (n = 12) (**C**) were assessed 3 days after injection. (**D**) The walking distances of the termite *R. chinensis* after 3 days of dsRNA injections during a 48 h experimental period. (**E**,**F**) The walking distances of the termite *R. chinensis* 3 days after injecting dsRNA during subjective day (**E**) and night (**F**). (**G**–**I**) The circadian rhythms of locomotor activities of the termite *O. formosanus* injected with ds*GFP* (n = 18) (**G**), ds*Cry2* (n = 18) (**H**), or ds*Per1* (n = 18) (**I**) were assessed 3 days after injection. (**J**) The walking distances of the termite *O. formosanus* after 3 days of dsRNA injections during a 48 h experimental period. (**K**,**L**) The walking distances of the termite *O. formosanus* 3 days after injecting dsRNA during subjective day (**K**) and night (**L**). The rhythmicity of locomotor activity was analyzed using the cosinor procedure (**A**–**C**,**G**–**I**). The data in the figures are mean ± SEM, and different letters express significant differences according to Tukey’s HSD test (*p* < 0.05) (**D**–**F**,**J**–**L**). Gray and black bars represent subjective day and subjective night, respectively (**A**–**C**,**G**–**I**).

The injection of ds*GFP* (control) did not significantly affect the circadian rhythms of locomotor activity under DD conditions in the termite *R. chinensis* (*p* < 0.001) (Figure 6A). However, the injection of ds*Cry2* or ds*Per1* disrupted the circadian rhythms of locomotor activity (ds*Cry2*, *p* = 0.152; ds*Per1*, *p* = 0.083) (Figure 6B,C). In addition, the injection of ds*Cry2* significantly decreased locomotor activity compared with the control in the termite *R. chinensis* (*ANOVA*, F_(2,32)_ = 4.34, *p* < 0.05) (Figure 6D). In the termite *O. formosanus*, the injection of ds*GFP* did not significantly impact the circadian rhythms of locomotor activity under DD conditions (*p* < 0.001) (Figure 6G), but the injection of ds*Cry2* or ds*Per1* disrupted the circadian rhythms of locomotor activity under DD conditions (ds*Cry2*, *p* = 0.128; ds*Per1*, *p* = 0.230) (Figure 6H,I). Moreover, the injection of ds*Cry2* or ds*Per1* significantly decreased locomotor activity compared with the control in the termite *O. formosanus* (*ANOVA*, F_(2,51)_ = 21.68, *p* < 0.001) (Figure 6J).

In addition, during the subjective day, the injection of ds*Cry2* or ds*Per1* significantly decreased locomotor activity compared with the control under DD conditions in the termite *R. chinensis* (*ANOVA*, F_(2,32)_ = 13.76, *p* < 0.001) (Figure 6E), but during the subjective night, the injection of ds*Cry2* or ds*Per1* had no effect on locomotor activity as compared to the control (*ANOVA*, F_(2,32)_ = 0.17, *p* = 0.849) (Figure 6F). For the termite *O. formosanus*, the injection of ds*Cry2* or ds*Per1* significantly decreased locomotor activity during the subjective day and night compared with the control under DD conditions (*ANOVA*, Day, F_(2,51)_ = 45.56, *p* < 0.001; Night, F_(2,51)_ = 6.62, *p* < 0.01) (Figure 6K,L).

## 4. Discussion

Even though circadian rhythms are self-sustaining under constant conditions, organisms (insects and mammals) nevertheless respond to environmental stimuli, particularly illumination intensity and duration [41,42,43]. In this study, our results showed that the two termites *R. chinensis* and *O. formosanus* exhibited no clear circadian rhythms of locomotor activity under LL and LD conditions, which is different to the pattern observed in other insects and mammals [18,44]. In addition, since these two termite species have adapted to dark, underground environments, light may be a stressful environment for subterranean termites, which indicates that light conditions impair the circadian rhythms of locomotor activity in termites. Thus, subterranean termites have evolved different circadian rhythms of locomotor activity in response to their living environment. Surprisingly, under DD conditions, the termites *R. chinensis* and *O. formosanus* exhibited clear circadian rhythms of locomotor activity. Similarly, the cockroach *Periplaneta americana* exhibited a clear circadian rhythm of locomotor activity under complete darkness [18]. The subterranean vole *Lasiopodomys mandarinus* (eusocial mammal) also exhibited a clear circadian rhythm of locomotor activity under DD conditions [44]. These observations reflect the adaptation of subterranean termites, cockroaches, and voles to dark underground environments. Because the circadian rhythms of locomotor activity are exhibited even in constant conditions, we suspect that the endogenous circadian clock is important for the regulation of the circadian rhythms of locomotor activity in termites.

In insects and mammals, the expression of clock genes fluctuated rhythmically in a 24 h period [24,45]. In this study, the *Cry2* levels of two termite species exhibited a clear circadian rhythm, with higher levels during subjective day and lower levels during subjective night under DD conditions, which exactly coincided with the circadian rhythm of locomotor activity in two termite species. *Per1* levels showed a similar trend to *Cry2*, exhibiting a clear circadian rhythm. The *Cry* and *Per* genes are established clock core complex molecules [46,47]. We conclude that *Cry2* and *Per1* genes may play a critical role in the circadian rhythm of locomotor activity in these two termite species.

Knockdown of *Cry2* or *Per1* significantly impaired the circadian rhythms of locomotor activity and markedly reduced locomotor activity under DD conditions in *R. chinensis* and *O. formosanus*. Analogously, knockdown of *Cry2* severely disrupted the circadian rhythms of locomotor activity under DD conditions in the cockroach *B. germanica* [18]. Silencing of *Per* in the cricket *G. bimaculatus* completely disrupted the circadian rhythm of locomotor activity in constant darkness [21]. In mammals, *Cry2* mutation caused a shortened circadian period and impaired behavioral rhythms under a light–dark cycle in mice [19]. We suggest that the two core clock genes *Cry2* and *Per1* can regulate the circadian rhythms of locomotor activity under DD conditions in termites. Furthermore, knockdown of *Cry2* or *Per1* in *R. chinensis* and *O. formosanus* markedly reduced locomotor activity during the day, which indicates that *Cry2* and *Per1* are essential for the maintenance of termite circadian rhythms and their locomotor behavior.

## 5. Conclusions

Taken together, our study delivers evidence that the two termites *R. chinensis* and *O. formosanus* exhibit clear circadian rhythms of locomotor activity under DD conditions. However, under LD and LL conditions, complete loss of circadian rhythms of locomotor activity was observed in these two termites. Subterranean termites’ foraging activities mainly occur during the subjective day. Strikingly, the clock genes *Cry2* and *Per1* display cyclical expression in the heads of termites under DD conditions. In addition, knockdown of *Cry2* and *Per1* disrupted the circadian rhythms of locomotor activity in the two termites, which suggests that *Cry2* and *Per1* participate in termite circadian rhythms under DD conditions. Thus, this study contributes to a better understanding of circadian clock mechanisms in subterranean insects.

## Data Availability

The data supporting the results may be found either in the manuscript or in the supplementary information.

## Supplementary Materials

The following supporting information can be downloaded at https://www.mdpi.com/article/10.3390/insects15010001/s1, Figure S1: The patterns of *Cry2* and *Per1* gene expression in the two termite species; Table S1: The distribution of the four colonies of *R. chinensis* for each experiment; Table S2: The distribution of the seven colonies of *O. formosanus* for each experiment; Table S3: Primers used for qRT-PCR analyses; Table S4: Primers used for cloning the dsRNA template.
