# Supplementary material for: Circadian Rhythms of Locomotor Activity Mediated by Cryptochrome 2 and Period 1 Genes in the Termites Reticulitermes chinensis and Odontotermes formosanus
Source: Insects. 2023 Dec 19;15(1):1. doi: 10.3390/insects15010001 (PMC10816429; doi:10.3390/insects15010001)
Supplement: Supplementary file 1 [file insects-15-00001-s001.zip › insects-2745667-SI.pdf]

Supplementary information

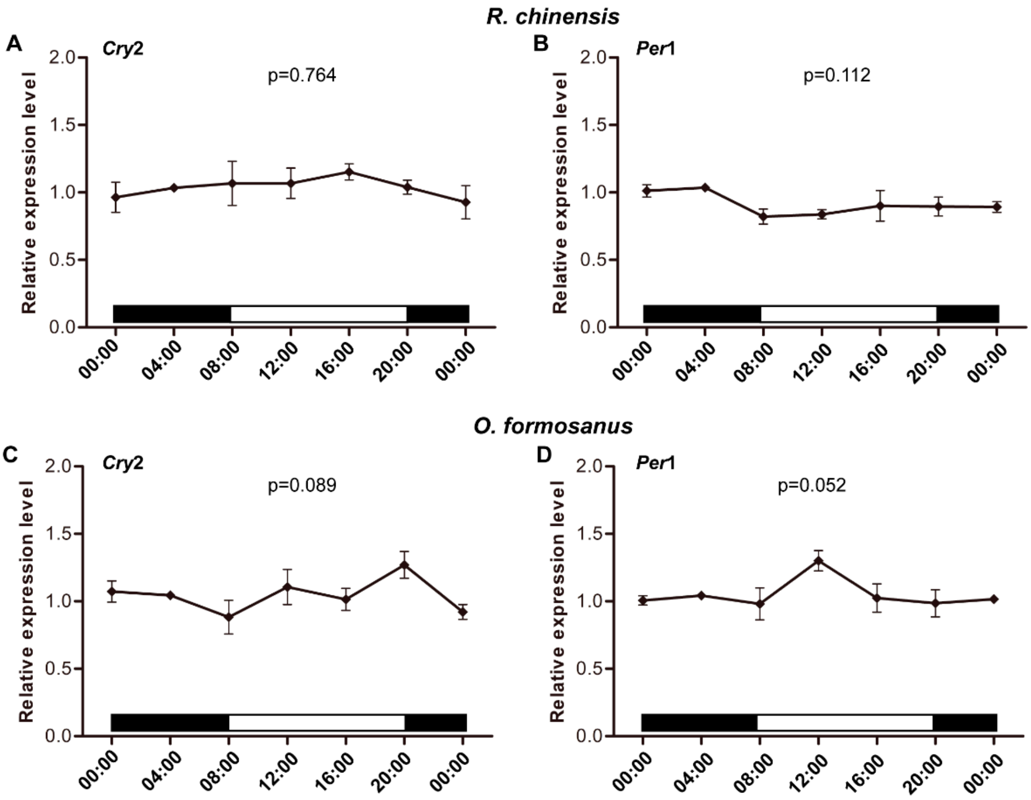

Supplementary Figure 1. The patterns of *Cry2* and *Per1* genes expression in the two termite species. (A) The patterns of *Cry2* expression in the termite *R. chinensis* under LD condition (n = 9). **(B)** The patterns of *Per1* expression in the termite *R. chinensis* under LD condition (n = 9). **(C)** The patterns of *Cry2* expression in the termite *O. formosanus* under LD condition (n = 9). **(D)** The patterns of *Per1* expression in the termite *O. formosanus* under LD condition (n = 9). The data in the figures are the mean ± SEM, and different letters express significant differences according to Tukey's HSD test. The mRNA levels were normalized relative to the two termite species collected at 04: 00. White and black bars represent subjective day and subjective night, respectively.

Supplementary Table S1. The distribution of the four colonies of *R. chinensis* for each experiment*

Experiments	Replicates	Colonies
Circadian rhythms of locomotor activity	9-10	Number 1, 2, 3
Expression of *Per*1 and *Cry*2	8-9	Number 2, 3, 4
RNAi efficiency for *Per*1 and *Cry*2	4-7	Number 2, 3, 4

* All the four colonies of *R. chinensis* were collected from Shizi hill in Wuhan City, Hubei Province, China.

Supplementary Table S2. The distribution of the seven colonies of *O. formosanus* for each experiment*

Experiments	Replicates	Colonies
Circadian rhythms of locomotor activity	9-10	Number 1, 2, 3
Expression of *Per*1 and *Cry2*	8-9	Number 2, 3, 4
RNAi efficiency for *Per*1 and *Cry2*	4-7	Number 5, 6, 7

* All the seven colonies of *O. formosanus* were collected from Shizi hill in Wuhan City, Hubei Province, China.

Supplementary Table S3. Primers used for qRT-PCR analyses

Species	Gene Name	Orientation	(5′→3′) Primer Sequence
R. chinensis	*β-actin*	Forward	AGCGGTCACTCATTCCCTTG
		Reverse	ATTCCTGACGTACTGTCGCC
	Hsp 70	Forward	GCTAAGCGTCTTATTGGGCG
		Reverse	CTTTGGTTTTCCACCGTCGC
	Cry2	Forward	AGTTCTTTGCACATGGCCG
		Reverse	CGTGAAGGACTGAACTCTGCT
	Per1	Forward	CTTCAGCCAACCACAGCTCTA
		Reverse	TGGTTCCGCTTTCACGGATA
O. formosanus	*β-actin*	Forward	CTGGAGAAGTCATACGAGTTG
		Reverse	AGAAGGAAGGCTGGAACA
	NADH	Forward	TTGGTGAGATTGGTCTGCTG
		Reverse	ACAATGTGTAAGCCGCACTA
	Cry2	Forward	TCTTACCCGTGGCGATCTCT
		Reverse	AAGCGCACTGGACAATAGCA
	Per1	Forward	GGAACATTGCCATGGGGTCA
		Reverse	TCCTTGGCATAAAGGGGACAC

Supplementary Table S4. Primers used for cloning the dsRNA template

Species	Gene Name	Orientation	(5′→3′) Primer Sequence
R. chinensis	*Cry2*	Forward	GAGGTGTCATTTTAGGGCGA
		Reverse	TTCAAGGAATGGGGCACTAC
	Per1	Forward	ACGAAGAGCCCTCTGGGATA
		Reverse	GCAGCATTGGACTGAACGTC
	GFP	Forward	CTTGAAGTTGACCTTGATGCC
		Reverse	TGGTCCCAATTCTCGTGGAAC
O. formosanus	*Cry2*	Forward	TCGCCCTAAAATGACACCTC
		Reverse	CACCACGGGTAAGAAAGCAT
	Per1	Forward	CCTCATATTGAGGGAGGCAA
		Reverse	TGGGTTGTTGTACTGGCAAA
	GFP	Forward	CTTGAAGTTGACCTTGATGCC
		Reverse	TGGTCCCAATTCTCGTGGAAC
